# A Preliminary Study of the Comfort in Patients with Leukemia Staying in a Positive Pressure Isolation Room

**DOI:** 10.3390/ijerph17103655

**Published:** 2020-05-22

**Authors:** Wun-Yu You, Tzu-Pei Yeh, Kwo-Chen Lee, Wei-Fen Ma

**Affiliations:** 1 Department of Nursing, China Medical University Hospital, Taichung 40402, Taiwan; peggyyou0509@gmail.com (W.-Y.Y.); tzupeiyeh@mail.cmu.edu.tw (T.-P.Y.); rubylee@mail.cmu.edu.tw (K.-C.L.); 2 School of Nursing, China Medical University, Taichung 40402, Taiwan; 3 Brain Disease Research Center, China Medical University Hospital, Taichung 40402, Taiwan

**Keywords:** anxiety, fatigue, comfort, distress symptom, leukemia, isolation, oncology, positive pressure isolation room

## Abstract

*Background and Aim:* Patients with leukemia who are isolated in positive pressure rooms for infection prevention usually experience significant physical and psychological distress. This study aimed to examine changes in leukemia patients’ comfort level during chemotherapy in isolation wards. *Methods:* A longitudinal survey was conducted with measures which were repeated four times. Data were collected before chemotherapy, on the first and second week after receiving chemotherapy in positive pressure isolation rooms, and on the third week in the non-isolated hematology ward. Each patient received six questionnaires measuring demographic data, comfort status, functional status, fatigue related to cancer therapy, anxiety level, and distress symptoms. A mixed model with repeated measure analysis was used to examine the changing trajectories in physical and psychological health. *Results:* Twenty-one patients completed the study. During the process, the highest score for comfort level was shown before chemotherapy, and this decreased from the second week under isolation. Anxiety and uncertainty (*p* < 0.05) declined over time, and emotional states improved during the recovery period in the third and fourth weeks outside isolation. Physical well-being (*p* < 0.01), cancer-related fatigue (*p* < 0.05), hemoglobin (*p* < 0.01) and white blood cell count (*p* < 0.05) began to rise two weeks after chemotherapy. *Conclusion:* Comfort levels declined after chemotherapy until the third week of treatment. Anxiety, fatigue and distress symptoms varied across the four time points of chemotherapy from isolation to return to the non-isolated ward. Health care professionals should be aware of psychological symptoms when patients are in isolation rooms, and interventions for promoting a humanized environment, quality of life, and comfort should be considered and provided along with the treatment stages of chemotherapy.

## 1. Introduction

### 1.1. Background Related to Leukemia

In 2018, leukemia was identified as the 10th leading cause of cancer death in Taiwan [1]. Most patients with leukemia seek medical help after the occurrence of anemia, coagulopathy, or hyperthermia [2]. Among the different types of the disease, acute myelogenous leukemia poses the greatest therapeutic challenge as it is marked with the fastest progression and failure rate of treatments, especially for elderly patients. Responding to such active treatment may result in a short survival time about three months [3].

Unlike the methods with which patients with a solid tumor are treated, patients with leukemia usually receive chemotherapy as soon as they are diagnosed; therefore, they usually experience acute and severe side effects of chemotherapy due to blood cell aplasia [4]. The white blood cell count (WBC) may drop to its lowest about 7~14 days after the start of chemotherapy. This period is also the time when the immunity, anemia, and coagulation reach their lowest levels [5]. Isolation wards for patients with leukemia are designed with positive pressure and a special airflow to avoid the ingress of outside air into the isolation room. This may reduce the risk of airborne infection caused by changes in airflow when medical staff enter and exit [6,7]. Patients suffer from physical discomfort and fatigue from the diagnosis of the disease throughout the course of chemotherapy.

### 1.2. Discomfort of Leukemia Patients Undergoing Chemotherapy

Leukemia patients suffer from both physical and psychological discomforts during the entire process from the onset of the disease to the end of its treatment [8]. According to the study by Shephard and colleagues [9], no significant differences in symptoms were observed among 4655 patients receiving treatments for acute and chronic leukemia. They all suffered from fatigue, infection, weight loss, and severe discomfort, regardless of the chemotherapy treatments. Fatigue is the most common physical discomfort [5]. As defined by the National Comprehensive Cancer Network, cancer-related fatigue (CRF) refers to “a persistent, subjective sense of tiredness related to cancer or cancer treatment that interferes with usual functioning” [10]. Along with the huge impacts of disease symptoms and the side effects of treatments, patients with leukemia constantly experience physical discomforts such as gum bleeding, declining appetite, mucositis, sleep disorder, and persistent fatigue [11]. Therefore, health care providers need to understand the changes in the comfort level of patients with leukemia undergoing chemotherapy.

Psychological discomfort, on the other hand, is closely correlated with anxiety and depression [12]. Patients experience a profound and persistent sense of uncertainty about one’s future and fear of death, and those feelings are exacerbated by body and appearance changes due to the disease and treatment, generating escalating anxiety and depression [13]. The growing severity of the condition and dwindling control over the disease can set off a surge in negative emotions [14], which in turn may drastically dampen the will and ability of patients to battle leukemia when they are already affected by overwhelming CRF during the chemotherapy treatment.

All the discomforts make it extremely difficult for leukemia patients to cope with physical pain and psychological pressure [15]. More recently, Tinsley and colleagues [16] provided an empirical finding indicating that quality of life returned to stability at 1 month in patients with acute myeloid leukemia treated with intensive chemotherapy. Schumacher and colleagues [17] reported that, for leukemia patients, quality of life could be improved from the beginning of chemotherapy to the end of inpatient treatment. They also emphasized the role of the health environment in improving quality of life [17].

According to the theory of comfort developed by Kolcaba [18], there are three levels of comfort (relief, ease, and transcendence) including four dimensions (physical, psycho-spiritual, social, and environmental). Human beings are usually able to adapt to external stimuli to maintain comfort in the aforementioned four contexts, which need to be assessed to explore patients’ comfort status and affecting factors. Promoting an understanding of the factors which affect the comfort of patients with leukemia during chemotherapy can help healthcare professionals to provide appropriate coping strategies and interventions for alleviating discomforts and promoting engagement in the therapy of leukemia patients. However, only a few studies have been conducted to examine the comfort status and the factors affecting the comfort of leukemia patients during the chemotherapy process.

### 1.3. Study Aim

This study aimed to examine the fluctuations in the overall comfort of leukemia patients during the chemotherapy process under isolation circumstances. The study focused on tracing the changes in the patients’ comfort levels and distress associated with chemotherapy-induced symptoms, CRF, feelings of uncertainty, and anxiety. Meanwhile, the factors affecting comfort in patients with leukemia during the different stages of the chemotherapy process were also explored.

## 2. Materials and Methods

### 2.1. Research Design and Participants

A longitudinal survey with purposive sampling was performed. The data were collected 3 days before the chemotherapy (T0), at the first follow-up on days 6~8 of the treatment course (T1), at the second follow-up on days 13~15 (T2), and at the third follow-up on days 20~22 (T3), respectively. T0 to T2 data were collected in the isolation room, and T3 data were collected in a non-isolated room in the hematology ward. Each participant answered six questionnaires from T0 to T3.

The participants were recruited from the hematology ward of a medical center in central Taiwan based on the following inclusion criteria: diagnosed with leukemia by a physician, aged 20 years or older, and capable of communicating in Mandarin or Taiwanese. Patients who were confused, unable to communicate, and who were receiving both chemotherapy and radiotherapy were excluded.

### 2.2. Data Collection and Ethics Approval and Consent to Participate

After receiving an approval letter from the Institutional Review Board of China Medical University Hospital (No. CMUH103-REC3-087, CR & CR-1), the researcher began data collection. During recruitment, the study purpose, data collection methods, and relevant issues were explained to the patients who were approached. Their right to receive appropriate medical care was assured and remained intact no matter whether they decided to participate in this study. After signing an informed consent form, participants were asked to receive both a physical examination and questionnaire surveys at the four different time points (from T0 to T3). During the study process, the participants were able to decide to withdraw for any reason or without cause.

### 2.3. Measures

Six instruments were used in this study. As they were receiving chemotherapy for leukemia, the participants regarded as patients were in very poor or even life-threatening condition. Measuring physical status was therefore particularly important. In addition to one instrument regarding comfort, five more instruments were used to collect demographic data and psychological uncertainty, anxiety, physical symptom distress, and physical CRF-related factors.

#### 2.3.1. Demographic Inventory to Measure Personal Data

The Demographic Inventory incorporates five items: age, gender, education level, marital status, and the time point from diagnosis to chemotherapy treatment. In addition to subjective feelings, physiological data may provide more objective evidence for analysis. The personal data in our study accordingly further included hemoglobin, WBC, albumin, body mass index (BMI), body temperature, heart rate, respiratory rate, systemic blood pressure (SBP), and diastolic blood pressure (DBP; each was measured prior to the participant answering a questionnaire.

#### 2.3.2. Shortened (28-Item) General Comfort Questionnaire to Measure Comfort

The 28-item shortened GCQ developed by Kolcaba in 2003 was used to measure comfort [19]. The questionnaire measures comfort in four contexts (physical, psychospiritual, social, and environmental) and at three levels (relief, ease, and transcendence) based on a 6-point Likert-type scale ranging from “Strongly Disagree” to “Strongly Agree”. A higher score indicates a higher level of comfort. The internal consistency of the questionnaire was 0.88, and the correlation between subscales ranged from 0.51 to 0.62 [19].

#### 2.3.3. Mishel Uncertainty in Illness Scale to Measure Uncertainty

The Mishel Uncertainty in Illness Scale, developed by Mishel (1988) and translated into Chinese by Sheu and Hwang [20], includes 25 items with a Likert scale ranging from the one-point “Strongly Disagree” to the five-point “Strongly Agree”. A higher score indicates a greater degree of uncertainty. The Cronbach’s alpha of the scale was 0.91 and the criterion validity was 0.57 (*p* < 0.001) when compared with the Situational Anxiety Scale.

#### 2.3.4. Chinese Mandarin State–Trait Anxiety Inventory Y to Measure Anxiety

Spielberger’s State–Trait Anxiety Inventory-Y was translated from the English version into Chinese Mandarin by Ma and colleagues [21]; the CMSTAI-Y incorporates a 20-item state anxiety inventory and a 20-item trait anxiety subscale. The scale measures state anxiety on a four-point Likert-type scale ranging from “Strongly Disagree” to “Strongly Agree”. A higher score indicates a lower level of anxiety. In this study, only state anxiety was measured and used. A Cronbach’s alpha value of 0.92 and a 2-week test–retest reliability of 0.76 were reported for the state anxiety subscale, and its high correlation with the Chinese Hamilton Anxiety Rating Scale indicated fine criterion validity (*r* = 0.69, *p* < 0.001, 95% Confidence interval = 0.509~0.072) [21].

#### 2.3.5. Symptom Distress Scale to Measure Physical Symptom Distress

The Symptom Distress Scale developed by McCorkle and Young [22] and translated into Chinese by Tang [23] was adopted to measure distress symptoms in this study. The scale incorporates 13 items measured by a five-point Likert scale. A higher score indicates a higher level of physical distress. The Cronbach’s alpha, expert validity, and internal consistency of the scale were 0.85, 80%, and 0.78~0.83, respectively [24].

#### 2.3.6. Functional Assessment of Cancer Therapy–Fatigue Subscale to Measure CRF

The Functional Assessment of Cancer Therapy–Fatigue Subscale (FACT-F) developed by Yellen, Cella, Webster, Blendowski, and Kaplan [25] was translated into Chinese by Eremenco and colleagues [26]; it measures the overall well-being and CRF of cancer patients. Among the 40 items on the full scale, seven are for physical well-being, seven for emotional well-being, six for social well-being, seven for functional well-being, and 13 for fatigue. A higher overall score denotes a better quality of life and lower level of fatigue. The Cronbach’s alpha of this scale was 0.95 and its test–retest reliability was 0.87 [25]. The subscales reported an internal consistency of 0.95~0.96 and a test–retest reliability of 0.93~0.95 [25].

### 2.4. Statistical Analysis

The SPSS software package version 22.0 (SPSS, Chicago, IL, USA) was used to analyze the collected data. Descriptive statistics were adopted to present the results of the demographic data, physical assessments, and self-report questionnaires. Stepwise multiple regression analysis was performed to study the influences of variables on comfort level in each stage during the course of chemotherapy treatment. A mixed model with repeated measure analysis was used to examine the changing trajectories in comfort and other study variables during the chemotherapy process.

## 3. Results 

### 3.1. Demographic and Physical Assessments

Out of the 35 patients approached during recruitment, 23 agreed to participate and 14 declined due to a lack of motivation (*n* = 3), fear of being too weak to participate (*n* = 10), or for no specified reason (*n* = 2). Two of the participants who agreed to participate were unable to complete the study due to death with acute myocardial infarction and fatigue. The remaining 21 participants included seven women and 14 men with a mean age of 45.24 (SD = 15.71) and an average length of education of 13.10 (SD = 3.18) years; 14 (66.7%) were married and six were single. While all participants received chemotherapy treatment, 65% of the study participants (*n* = 14) were newly diagnosed with acute leukemia. In total, 16 (76.2%) participants were religious. The average time period from diagnosis to chemotherapy treatment was 4.76 (SD = 0.57) months.

### 3.2. Physical Condition Measures

Overall physical condition appeared to deteriorate over time during the course of chemotherapy, with DBP rising and SBP and BMI declining. WBC, hemoglobin levels and albumin continued to drop and reached their lowest points at T2 during the second week of chemotherapy. The mean values and the trajectories of physical conditions analyzed by the mixed model with repeated measures are reported in Table 1.

### 3.3. Trajectory of Well-being and Comfort Status

The mean values of comfort showed a steady decline over the course of chemotherapy, marked with an improvement at T3. For measures of well-being, there was no consistency in the appearance of the highest score, as this was at the beginning of treatment (T0) for state anxiety and CRF and at T3 for functional and emotional well-being. The details are reported in Table 2. Moreover, the results identified statistical significance in the trajectories of WBC, hemoglobin, and the physical well-being by the analysis of the mixed model with four repeated measures.

### 3.4. Factors Affecting Comfort in Four Time Points of Chemotherapy

Stepwise regression was used to analyze the factors affecting comfort in the four different time points of the chemotherapy process. Anxiety appeared to significantly affect comfort in the early time point of chemotherapy, while uncertainty and functional well-being emerged as significant comfort-affecting factors in the second week during the chemotherapy treatment (T2); 75.0% of the variance was explained. By the third week (T3), the duration from diagnosis to chemotherapy treatment and hemoglobin together explained 67.9% of the variance (Table 3).

## 4. Discussions

### 4.1. Innovation of the Research

The results showed that comfort, anxiety, fatigue, and distress symptoms among patients with leukemia varied across the four time points of chemotherapy treatment in isolated and non-isolated environments. Comfort levels fluctuated over the course of the treatment, reaching their highest level before treatment and undergoing a continuous decline until the second week, as they were influenced by both the physical conditions and psychological status of the patients with leukemia under isolation protection in this study. To the best of our knowledge, our study, which is based on the theory of comfort [18], is the first to provide evidence related to the comfort level of Taiwanese leukemia patients during chemotherapy treatment under isolation. However, our study focused on physically weak inpatients receiving chemotherapy for life-threatening leukemia; it is difficult for our study to obtain outcomes related to higher levels of comfort, such as ease and transcendence. Instead, the results tend to be better applicable to the lower comfort level of “relief” in patients with serious physical and psychological discomforts.

In our study, patients started at T1 to experience functional decline and distress symptoms in daily life. The lowest scores observed at T2 for physical and functional well-being and fatigue, together with the total FACT-F scores, indicate an overall physical condition affected directly by chemotherapy and which showed no improvement until the second week after the treatment start. Distress symptoms, on the other hand, were severe at the beginning of the chemotherapy treatment and began to be mitigated at the third week. Meanwhile, the WBC count dropped to its lowest as the immunity, anemia, and coagulation of the participants reached their nadir [4,5]. These findings are similar to those reported in the study by Tinsley and colleagues [16]; i.e., that the quality of life was improved at 1 month for patients with acute myeloid leukemia treated with intensive chemotherapy.

Psychological status exerted a greater influence on leukemia patients’ comfort before than after chemotherapy as anxiety declined over time and emotional state improved during the recovery period. Similar findings were also reported by Hu and colleagues [27], as well as by Morrison and colleagues [28]: i.e., lower scores in emotional well-being or higher ones in negative emotions were observed in leukemia patients before chemotherapy. Conducting emotion assessments and sharing negative feelings in T0 should be considered to be helpful to obtain a better understanding about the factors which can trouble patients. Sufficient information and knowledge should be provided several times before and after chemotherapy, with flexible opportunities for discussions and verification [29,30].

The impacts of an isolation environment on patients’ quality of life and comfort must not be overlooked. Health care providers need to be aware of the physical and psychological symptoms in leukemia patients before chemotherapy under isolation [31,32]. At T2, attention should be directed to physical and mental well-being, which are bound to be heavily impacted by chemotherapy-related distress symptoms. While limiting the number of visitors and/or implementing other protective isolation measures are necessary to protect leukemia patients from infection [33,34], they may give rise to feelings of aloneness and helplessness. This finding of our study is echoed in a survey on 17 breast cancer patients receiving chemotherapy [35], in whom feelings of isolation and alienation were found to be able to trigger extreme distress and panic; in turn, this may prevent patients from completing the entire course of chemotherapy treatment [36]. It has also been emphasized by Buffoli and colleagues [37] that the humanization of hospital environments can improve patients’ quality of life and comfort. Maintaining social activities by allowing the use of a cell phone, computer, or iPad to reduce the feeling of alienation caused by protective isolation is thus recommended.

As indicated by our study, patients with leukemia experienced a gradual decline in comfort with the progress of the chemotherapy treatment until the third week, and their reducing white blood cell count further put them at risk. In contrast, anxiety and distress symptoms appeared to change in the opposite direction. A general improvement was observed in the final stage of recovery, when emotional and functional well-being appeared to be positively correlated with general comfort. Similar results concerning the relationship between emotion and physical function can be found in the study by Klepin [13]. Overall, comfort improves gradually with the resurgence of WBC and the mitigation of distress symptoms during the third week of chemotherapy. A continuous assessment of the physical well-being and fatigue status of leukemia patients should be made to help increase comfort and decrease possible repeated hospitalization [32,38]. Corresponding interventions based on these findings can be developed by clinical health care providers to help improve the overall comfort of leukemia patients receiving chemotherapy in an isolated environment [39].

### 4.2. Limitation and Further Research

Our study experienced difficulty in recruiting leukemia patients. Some of them were too anxious and depressed (probably due to fear of death) [29] to respond to our recruitment. Some were too weak to complete the questionnaires. Including four time points of data collection also made the process into a challenging task, especially when participants were moved into isolation rooms for infection prevention after the first week of chemotherapy. While patients were isolated, every single item, including the questionnaire, needed to be sterilized before being distributed to participants. While our study found a general decline in comfort during the treatment process, a larger sample size is needed in future studies to make the research results more generalizable and convincing. Moreover, only data during the participants’ stay in the hospital were collected; a longer longitudinal survey may provide more in-depth and detailed information.

## 5. Conclusions

Patients with leukemia suffer from both physical and psychological discomforts that are exacerbated by the isolated environment while chemotherapy is administered. The comfort levels of this group of patients and factors affecting their comfort have been understood; this work’s longitudinal survey covering the entire chemotherapy regimen made this study particularly unique. In terms of the lack of related studies, and with the belief that improving leukemia patients’ comfort levels during chemotherapy follows the essential purpose of humanized healthcare, this study conducted a longitudinal survey with purposive sampling to trace the changes in the comfort level of 21 leukemia patients receiving chemotherapy in isolation rooms. Data were collected before chemotherapy and at the first, second, and third weeks of chemotherapy. Comfort fluctuated throughout the process and appeared to be at its highest before treatment and underwent a decline until the second week. Anxiety declined over times and emotional state improved during the recovery period. Physical well-being, cancer-related fatigue, and WBC count began to rise after chemotherapy in 2 weeks. Distress symptoms were severe at the beginning of treatment and started to show an improvement at the third week. Health care providers are encouraged to pay close attention to the fluctuations of these symptoms to provide appropriate and timely interventions to improve the comfort status of patients with leukemia during the isolated period of chemotherapy.

## Figures and Tables

**Table 1:** Mean (SD) and trajectories of physical assessment by mixed model analysis.

Physical Assessment	T0/Mean (SD)	T1/Mean (SD)	T2/Mean (SD)	T3/Mean (SD)	*F*	*p*
Body Mass Index	23.33 (4.56)	22.18 (4.56)	22.21 (4.97)	21.99 (4.95)	0.353	0.787
Systemic Blood Pressure	121.67 (18.28)	115.26 (14.89)	116.68 (15.73)	114.68 (30.44)	0.575	0.630
Diastolic Blood Pressure	68.48 (8.86)	72.63 (10.57)	70.32 (9.10)	76.32 ** (9.20)	2.717	0.058
White Blood Cell	5594.74 (8722.22)	2482 (4255)	626 * (1220)	4057 (4213)	6.135	0.002
Hemoglobin	10.18 (1.75)	9.31 (1.80)	8.38 ** (1.20)	8.58 ** (0.75)	6.156	0.002
Albumin	3.84 (0.87)	3.45 (0.54)	3.26 * (0.38)	3.77 (0.61)	1.88	0.209

* *p* < 0.05; ** *p* < 0.01; T0 = before chemotherapy; T1 = Days 6¨C8; T2 = Days 13¨C15; T3 = Days 20¨C22.

**Table 2:** Mean (SD) and trajectories of comfort, anxiety, cancer-related fatigue and distress symptoms by mixed model analysis.

Variables	T0/Mean (SD)	T1/Mean (SD)	T2/Mean (SD)	T3/Mean (SD)	*F*	*p*
Comfort	109.76 (18.54)	109.58 (15.40)	106.74 (19.54)	109.69 (15.29)	0.120	0.948
Uncertainty	71.90 (12.36)	67.84 (13.39)	67.05 (14.44)	61.42 * (12.08)	2.478	0.076
Anxiety	50.76 (11.08)	49.79 (8.75)	48.84 (10.62)	45.84 (9.19)	0.955	0.425
FACT-F ^a^	97.71 (17.32)	92.79 (17.14)	83.05 * (26.09)	90.58 (19.29)	1.529	0.221
PWB	18.00 (4.66)	15.21 (5.71)	12.16 ** (7.31)	16.00 (6.12)	3.162	0.034
SWB	18.90 (2.89)	18.37 (4.19)	18.21 (5.31)	17.79 (4.95)	0.291	0.832
EWB	15.95 (4.72)	16.89 (3.54)	17.16 (5.38)	18.05 (4.10)	0.772	0.517
FWB	11.57 (6.88)	12.47 (5.43)	10.95 (7.26)	12.53 (5.20)	0.272	0.845
CRF	33.29 (8.98)	29.84 (9.39)	24.58 * (12.86)	26.21 * (10.81)	2.767	0.054
DS	25.90 (8.04)	27.53 (5.90)	30.47 (8.23)	27.84 (7.73)	1.072	0.373

* *p* < 0.05; ** *p* < 0.01; T0 = before chemotherapy; T1 = Days 6¨C8; T2 = Days 13¨C15; T3 = Days 20¨C22. CRF = cancer-related fatigue; PWB = physical well-being; SWB = social well-being; EWB = emotional well-being; FWB = functional well-being; ^a^ FACT-F = total items of Functional Assessment of Cancer Therapy–Fatigue; DS = distress symptoms.

**Table 3:** Influences of variables on comfort during four stages of chemotherapy.

Stage	Variables	β	*T*	*R*	*R* ^2^	Adjusted *R*^2^	*F*
1	Intercept	90.113	3.896 **	0.892	0.796	0.759	22.046 ***
	Anxiety	−0.527	−2.234 *				
	Emotional Well-being	1.608	2.863 *				
	Physical Well-being	1.280	2.452 *				
2	Intercept	82.290	4.236 **	0.944	0.892	0.861	28.875 ***
	FACT-F	0.531	4.635 ***				
	Diagnosis-to-Treatment Duration	14.557	5.771 ***				
	Diagnosis-to-Treatment Duration	−0.754	−3.537 **				
	Age	0.224	2.256 *				
3	Intercept	162.775	12.948 ***	0.882	0.778	0.750	27.994 ***
	Uncertainty	−0.989	−6.020 ***				
	Functional Well-Being	0.938	2.870 *				
4	Intercept	117.627	4.572 ***	0.856	0.733	0.679	13.697 ***
	FACT-F	0.600	5.489 ***				
	Diagnosis-to-Treatment Duration	10.830	2.979 ***				
	Hemoglobin	−7.764	−2.820 *				

* *p* < 0.05, ** *p* < 0.01, *** *p* < 0.001; FACT-F = Functional Assessment of Cancer Therapy–Fatigue.
